# MUS81 Inhibition Enhances the Anticancer Efficacy of Talazoparib by Impairing ATR/CHK1 Signaling Pathway in Gastric Cancer

**DOI:** 10.3389/fonc.2022.844135

**Published:** 2022-04-11

**Authors:** Tao Wang, Peng Zhang, Chengguo Li, Weizhen Liu, Qian Shen, Lei Yang, Gengchen Xie, Jie Bai, Ruidong Li, Kaixiong Tao, Yuping Yin

**Affiliations:** Department of Gastrointestinal Surgery, Union Hospital, Tongji Medical College, Huazhong University of Science and Technology, Wuhan, China

**Keywords:** gastric cancer, MUS81, PARP inhibitor, BRD4 inhibitor, DNA damage

## Abstract

MUS81 is a critical endonuclease involved in heterodimer formation with Eme1/Mms4 and an important DNA damage repair regulatory molecule. Our previous study suggested that MUS81 was overexpressed and its high expression was positively correlated with gastric cancer metastasis. However, the therapeutic potential of targeting MUS81 in gastric cancer requires further exploration. Therefore, in this study, the Cancer Genome Atlas (TCGA) data were analyzed and showed that MUS81 is a key regulator of cell cycle distribution and DNA damage repair in gastric cancer. *In vitro* and *in vivo*, MUS81 knockdown significantly enhanced the anticancer effect of the PARP inhibitor talazoparib. Mechanistically, MUS81 inhibition impaired the activation of the ATR/CHK1 cell cycle signaling pathway and promoted gastric cancer cells with talazoparib-induced DNA damage to continue mitosis. Moreover, addition of the bromodomain-containing protein 4 inhibitor AZD5153 increased the anticancer effect of talazoparib *via* MUS81 inhibition in gastric cancer cells, and this combination effect was largely impaired when MUS81 was knocked down. In conclusion, these data suggested that MUS81 regulated ATR/CHK1 activation, a key signaling pathway in the G2M checkpoint, and targeting MUS81 enhanced the antitumor efficacy of talazoparib. Therefore, AZD5153 combined with talazoparib may represent a promising therapeutic strategy for patients with MUS81 proficient gastric cancer.

## Introduction

Gastric cancer is one of the most prevalent forms of cancers and the third leading cause of cancer-related deaths worldwide (1). For early gastric cancer, radical surgical resection is currently the main treatment. However, among patients newly diagnosed with gastric cancer, 70% of them have advanced gastric cancer, and surgery combined with chemoradiotherapy is the standard treatment (2). Limited by the high heterogeneity of gastric cancer biology and genetics, the effect of traditional chemotherapy regimens for gastric cancer has less than optimal results (3). Therefore, more effective treatment strategies need to be explored.

PARP inhibitors block the single-strand break (SSB) repair pathway by targeting PARP, causing homologous recombination deficiency malignant tumors to be synthetically lethal (4, 5). MUS81 belongs to the Xpf family of structure-specific endonucleases (6). It acts as a DNA replication pressure sensor, regulating the DNA replication forks reactivation, further affecting important DNA damage repair pathways, such as cell non-homologous end joining and homologous recombination repair (HR) (6, 7). Inhibition of MUS81 expression in ovarian cancer, significantly impairs cell HR activity (8, 9). Our previous research showed that MUS81 was overexpressed in gastric cancer cells and might promote gastric cancer cell invasion and metastasis (10). However, the therapeutic value of targeting MUS81 requires further investigation.

In this study, whether MUS81 silencing enhances talazoparib sensitivity, was evaluated in gastric cancer, and the mechanism of MUS81 knockdown sensitizing the anticancer effect of talazoparib was characterized. Furthermore, it was observed that AZD5153 sensitized the anticancer effect of talazoparib in gastric cancer *via* down-regulating the expression of MUS81. This is the first report to show that AZD5153 sensitizes the efficacy of talazoparib in gastric cancer by increasing DNA damage and apoptosis.

## Materials and Methods

### Cell Lines, Cell Culture, and Antibodies

SGC7901 and BGC823 cells were purchased from the National Collection of Authenticated Cell Cultures of China. RPMI-1640 medium (Gibco, USA) containing 10% FBS (Gibco BRL, USA) was used to cultivate cells at 37°C in a 5% CO_2_ atmosphere.

The primary antibodies against histone H2AX (γ-H2AX)^S139^ (9718S), ATR (13934S), p-ATR^T1989^ (30632S), CHK1 (2360), p-CHK1^S317^ (12302S), p-Histone H3^Ser10^ (53348S), Ki67 (9449S), and GAPDH (5174S) were obtained from Cell Signaling Technology (MA, USA). Antibodies against cleaved PARP (ab32064) were obtained from Abcam (MA, USA). HRP-conjugated secondary antibodies (SA00001-1 and SA00001-2) and anti-MUS81 antibodies were purchased from Proteintech (Wuhan, China).

### Cell Survival Assay

SGC7901 and BGC823 cells (1×10^3^ cells/well) were seeded in 96-well plates, and then treated with 0 to 1 µmol/L of talazoparib for 5 d. After drug treatment, the cell proliferation rate was determined using the CellTiter™ AQueous assay (MTS, Promega). Ten microliters of CellTiter 96^®^ AQueous One Solution Reagent was added to each well. After incubating for 1 h at 37°C , the absorbance was read at 490 nm using a microplate reader. The cell proliferation rate was calculated using the following formula: (mean optical density (OD) treated well [−blank])/(mean OD control well [−blank]) × 100. Five technical replicates were prepared for each sample in three separate experiments.

### Clonogenic Assay

Cells (1×10^3^ cells/well) were seeded into 6-well plates and exposed to different concentrations of talazoparib (0 or 100 nmol/L) for 10 d. Cells fixation and staining were performed as previously described (11). Finally, cell clones in 6-well plates were evaluated by ImageJ. The experiments were carried out three times.

### Western Blot Analysis

Western blotting was performed as described previously (12). RIPA buffer (V900854, Sigma, MO, USA) containing phosphatase inhibitors (G2007, Servicebio, Wuhan, China) and protease (B14001, Bimake, TX, USA) was used to lyse cells. Equivalent proteins were separated by 7.5%–12.5% SDS-PAGE. Finally, protein bands were detected using the ECL detection reagent (Thermo Fisher Scientific, USA). The results were analyzed using ImageJ. Experiments were performed three times.

### Apoptosis and Cell Cycle Analysis

Cells (2 × 10^5^ cells/well) were seeded into 6-well plates and exposed to DMSO or talazoparib (1 µmol/L) for 3 d. To examine apoptosis, the cells were harvested by trypsinization and washed twice with PBS at 4°C . The cells were resuspended in 100 µL Annexin V binding buffer, and 5 µL 7-AAD and 5 µL allophycocyanin-Annexin V were added. After the cells were incubated for 15 min in the dark at room temperature, 100 µL Annexin V binding buffer was added, and the proportion of apoptotic cells was analyzed by flow cytometry.

Cell cycle detection was performed as described previously (13). These experiments were performed using three biological replicates.

### Immunofluorescence Assay

Coverslips were coated with 0.01% poly-L-lysine and exposed to DMSO, talazoparib (0.5 µmol/L), AZD5153 (1 µmol/L), or their combination after the cells were plated. After two days of treatment, immunofluorescence was performed by staining with primary antibodies against γ-H2AX (1:100), p-Histone H3 (1:1600), or p-CHK1 (1:800) as previously described (14). Then, the coverslips were incubated with DAPI and CY3-conjugated secondary antibodies. Finally, coverslips were scanned using a fluorescence microscope (Nikon, Tokyo, Japan). The reproducibility of the results was confirmed by at least three separate experiments.

### Cell Transfection and RNA Interference

Two different shRNA sequences were used for MUS81 knockdown. Lentivirus (GeneChem Co. Ltd., China) targeting the sequence of human MUS81 (shRNA #1: 5′-TACCAACAAACAGCAAGTGGG-3′, shRNA #2: 5′-CACGCGCTTCGTATTTCAGAA-3′) was purchased from GeneChem Co. Ltd. (Shanghai, China). The corresponding control shRNA sequence was 5′-TTCTCCGAACGTGTCACGT-3′. Lentiviral infection was conducted as described previously (15). Stable GC cell lines transfected with scramble shRNA, shRNA #1, or shRNA #2 were selected for 7 d with 4 µg/mL puromycin.

Two specific MUS81 siRNAs were purchased from RiboBio (Guangzhou, China). The negative control siRNA sequence is 5’-GGGUAUCGACGAUUACAAA-3’, and the sequences of siRNA #1 and siRNA #2 targeting MUS81 are 5’-TACCAACAAACAGCAAGTGGG-3’ and 5’-CACGCGCTTCGTATTTCAGAA-3’, respectively. Transient transfection was performed using Lipofectamine 2000 (Thermo Fisher Scientific, USA) and siRNAs for 24 h. Western blotting was used to verify the knockdown effect of MUS81.

### *In Vivo* Experiments

The Institutional Animal Care and Use Committee of Tongji Medical College, Huazhong University of Science and Technology approved all animal experiments in this study. A total of 20 five-week-old BALB/c-null mice were purchased from HFK Biotechnology (Beijing, China). SGC7901^shCtrl^ and SGC7901^shMUS81^ cells were resuspended in PBS to obtain a concentration of 1 × 10^7^ cells/mL, and then each mouse was subcutaneously injected 150 µL of the solution in the right flank. When the tumor volume reached 100 mm^3^, the mice were randomly divided into four groups: control, MUS81-knockdown, talazoparib-treated, and MUS81-knockdown plus talazoparib-treated groups (five mice per group). Mice in the talazoparib group was administered talazoparib (0.33 mg/kg) by gavage for 21 consecutive days. The control group was administered the same dose of DMSO and PBS by gavage. After treatment, the mice were sacrificed, tumors were collected and embedded in paraffin, and immunohistochemically stained.

### Immunohistochemistry

Immunohistochemistry was performed using an antibody against Ki67 or γ-H2AX at a dilution of 1:200 as previously described (16). A brown signal was defined as a positive reaction in the cells. The results of immunohistochemical staining using a mean score were compared, considering both the intensity of staining and the proportion of tumor cells; the staining intensity score was 3, strong; 2, moderate; 1, weak; and 0, negative. The positive cell frequency was defined as 4 (76%–100%), 3 (51%–75%), 2 (26%–50%), 1 (1%–25%), and 0 (none). Immunohistochemical staining was performed for each component and the final score was obtained *via* multiplying the score of staining intensity and the score of positive cell frequency. Scores 0–7 were defined as negative staining, and 8–12 were defined as positive staining.

### Statistical Analyses

Statistical analysis was performed using GraphPad Prism version 9. A *t*-test was performed for intergroup comparisons and data are presented as the mean ± standard deviation (SD). *P* values less than 0.05 were considered significantly different and all tests were two-tailed.

## Results

### MUS81 Was Highly Expressed and Enriched in DNA Damage Repair Pathways in Gastric Cancer

**Figure 1:**
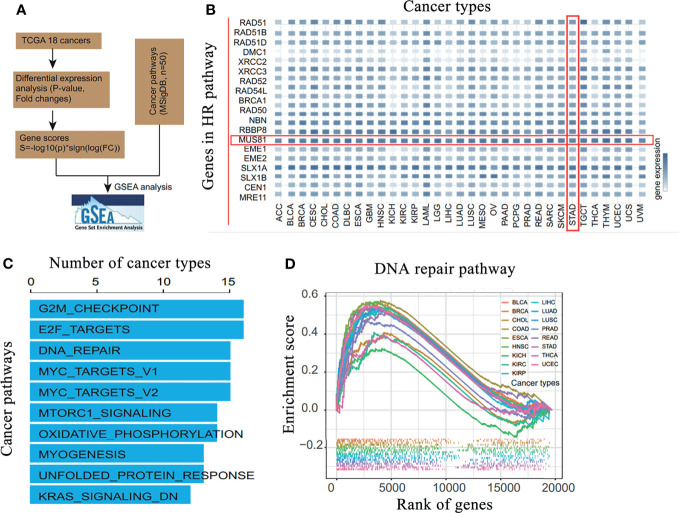
MUS81 is overexpressed in gastric cancer and is enriched in DNA damage repair pathways in malignant tumors. **(A)** Bioinformatics analysis process. **(B)** Analysis of the differential expression of DNA damage and repair-related molecules in 18 common malignant tumors in TCGA database suggests that MUS81 is overexpressed in gastric cancer. **(C)** Gene set enrichment analysis of MUS81-related enrichment signaling pathways in malignant tumors. **(D)** Enrichment of MUS81 in DNA damage repair pathways in malignant tumors.

Using TCGA database, the differential expression of DNA damage repair network-related molecules was analyzed in 18 common malignant tumors; MUS81 was significantly highly expressed in gastric cancer (**Figures 1A, B** **)**. Based on the gene set enrichment analysis in malignant tumors, MUS81 may play a critical role in many signaling pathways, such as the cell cycle regulation and DNA damage response pathways (**Figures 1C, D** **)**. These results suggested that targeting MUS81 is a potential treatment strategy in the clinical treatment of gastric cancer.

### MUS81 Knockdown Promoted Apoptosis and Anticancer Efficacy of Talazoparib *In Vitro*

**Figure 2:**
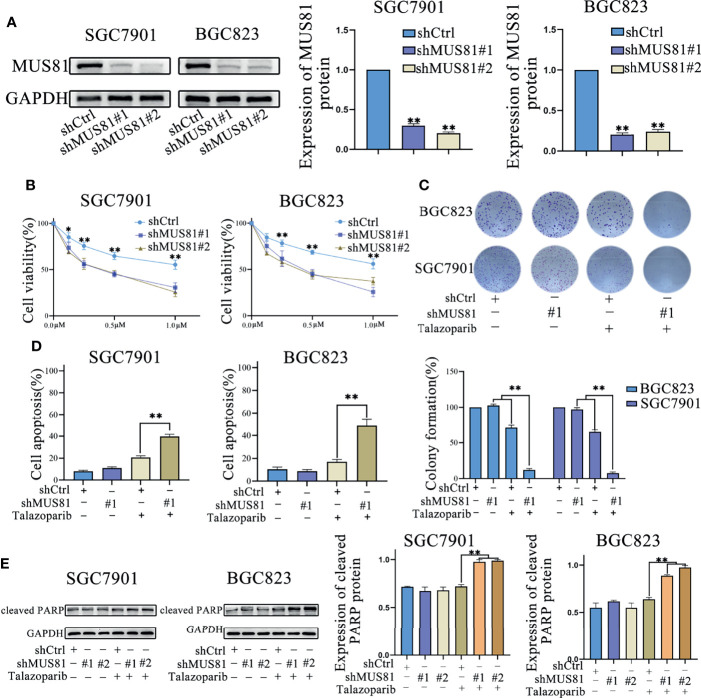
MUS81 knockdown promotes apoptosis and talazoparib anticancer efficacy *in vitro*. **(A)** Western blotting of MUS81 protein expression after transfection with the lentivirus expressing short hairpin RNA (shRNA) targeting the sequence of human MUS81 for 24 h. **P < 0.01. **(B, C)** Viability of SGC7901 and BGC823 cells transfected with lentivirus expressing shMUS81 #1, shMUS81 #2 or shCtrl, as determined using MTS and colony formation assays (t-test). *P < 0.05. **P < 0.01. **(D)** Cells treated with DMSO or talazoparib (1 µmol/L) for 3 d to evaluate apoptosis by flow cytometry (*t*-test). **P < 0.01. **(E)** Expression of apoptosis-related proteins in SGC7901 and BGC823 cells treated with DMSO or talazoparib (0.5 µmol/L) for 24 h, as evaluated by western blotting (*t*-test). **P < 0.01.

Next, SGC7901 and BGC823 cells were infected with the lentivirus vector (LV)-shMUS81. Western blotting was used to examine MUS81 protein levels in SGC7901 and BGC823 cells infected with LV-shMUS81 and LV-Ctrl. MUS81 protein levels were significantly decreased in MUS81 knockdown cells (**Figure 2A**).

MUS81 is an important DNA damage repair regulatory molecule and MUS81 deficiency impairs cell HR (8, 17). To explore whether down-regulation of MUS81 expression in gastric cancer cells affected the antitumoral efficacy of PARP inhibitors, MTS assay was performed and the data were analyzed to construct the dose-inhibition efficiency curves of the PARP inhibitor talazoparib in different groups. As shown in **Figure 2B**, cell proliferation of the LV-shMUS81 group was significantly decreased compared to that of the LV-Ctrl group in both SGC7901 and BGC823 cells, and the clone formation rate in the LV-shMUS81 group was significantly lower than that in the LV-Ctrl group after talazoparib treatment (71.69% ± 3.61% versus 12.38% ± 2.21% and 65.34% ± 3.22% versus 7.67% ± 1.53%, respectively, *P* < 0.01 for all) (**Figure 2C**).

To further confirm whether the antitumor effect of PARP inhibitors increased by MUS81 knockdown was due to increased apoptosis, flow cytometry was performed to measure the apoptosis rate of gastric cancer cells. Seventy-two hours after talazoparib treatment, a marked increase in apoptotic SGC7901 and BGC823 cells was revealed in the LV-shMUS81 group compared to that in the LV-Ctrl group (39.67% ± 2.08% versus 20.67% ± 1.50% and 48.69% ± 5.51% versus 17.34% ± 2.00%, respectively, *P* < 0.01 for all) (**Figure 2D**). In addition, western blotting also showed that the expression of cleaved PARP, a marker of apoptosis, increased significantly after exposure to talazoparib in the LV-shMUS81 group (**Figure 2E**).

### MUS81 Inhibition Pushed Cells Into Mitosis From G2M Checkpoint Arrest Induced by Talazoparib and Increased DNA Damage Caused by Talazoparib in Gastric Cancer Cells

**Figure 3:**
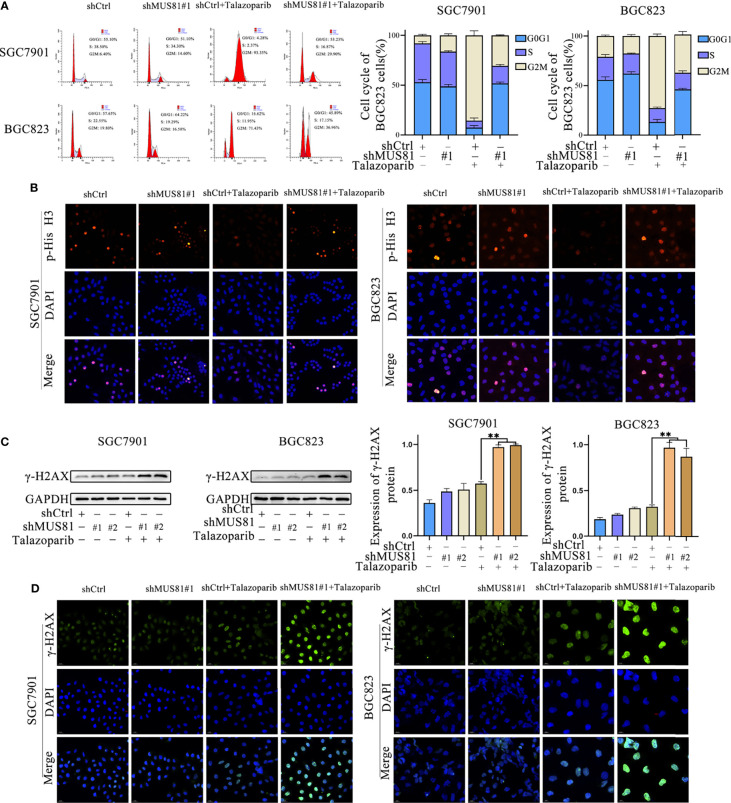
MUS81 inhibition may increase talazoparib-induced DNA damage in gastric cancer cells. **(A)** Cells treated with DMSO or talazoparib (1 µmol/L) for 3 d to evaluate cell cycle distribution by flow cytometry (*t*-test). **P < 0.01. **(B)** Immunofluorescence to detect the expression of p-Histone H3 (Ser10) in DMSO or talazoparib-treated (0.5 µmol/L) cells for 2 d. Magnification, 400×. **(C)** Western blot analysis of γ-H2AX expression after treatment with 0.5 µmol/L talazoparib for 24 h (t-test). **P < 0.01. **(D)** Immunofluorescence to detect the expression of γ-H2AX in DMSO or talazoparib-treated (0.5 µmol/L) cells for 2 d. Magnification, 400×. **P < 0.01.

To further investigate the mechanism by which MUS81 knockdown enhanced the sensitivity of SGC7901 and BGC823 cells to talazoparib, flow cytometry was performed for cell cycle analysis. Cell cycle analysis showed that talazoparib treatment induced significant G2M phase arrest in SGC7901 and BGC823 cells (**Figure 3A**). However, after stably transfecting with shMUS81, SGC7901 and BGC823 cells that were arrested by talazoparib in the G2M phase continued to mitosis, and the proportion of G0/G1 phase cells increased significantly (**Figure 3A**). To verify the effect of MUS81 knockdown on cell mitosis, the LV-shMUS81 and LV-Ctrl groups were stained for p-Histone H3 (Ser10), a mitosis marker, following treatment with talazoparib. MUS81 knockdown pushed gastric cancer cells with talazoparib-induced DNA damage into mitosis (**Figure 3B**).

Next, whether MUS81 deficiency impaired cellular DNA repair ability was tested. In this study, a DNA damage model was established using talazoparib. γ-H2AX expression was significantly increased after talazoparib treatment in LV-shMUS81 cells (**Figures 3C, D** **)**. These data indicated that MUS81 knockdown increased talazoparib-induced DNA damage in gastric cancer cells.

### MUS81 Deficiency Impaired ATR/CHK1 Activation Induced by Talazoparib in Gastric Cancer

**Figure 4:**
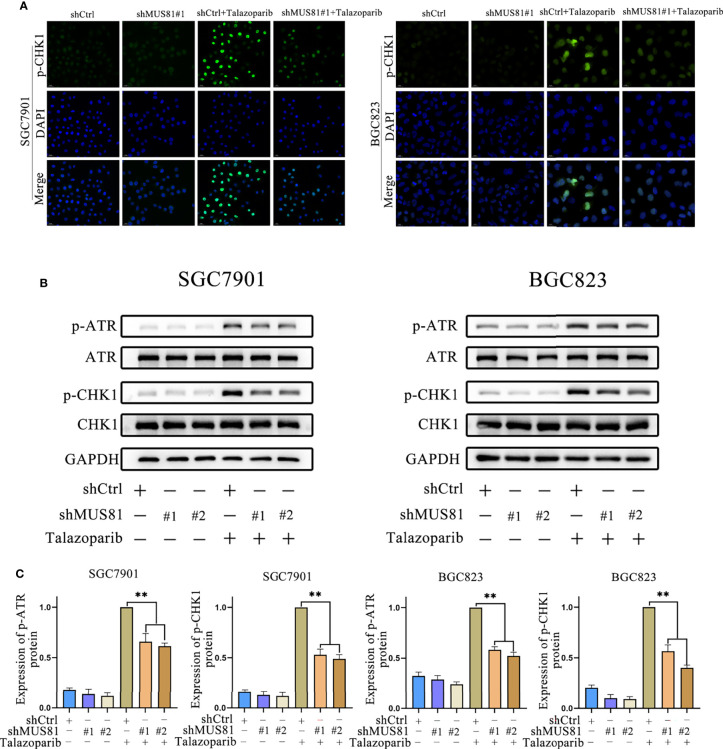
MUS81 deficiency impairs ATR/CHK1 activation induced by talazoparib in gastric cancer. **(A)** Immunofluorescence to detect p-CHK1 expression in talazoparib-treated (0.5 µmol/L) cells for 2 d. Magnification, 400×. **(B, C)** Western blot analysis examining the expression of p-ATR and p-CHK1 after treatment with 0.5 µmol/L talazoparib for 24 h (*t*-test). **P < 0.01.

Having observed a defective G2M phase checkpoint in MUS81-deficient cells, whether MUS81 deficiency impaired the DNA damage checkpoint signaling pathway was further examined. Because CHK1 is a key G2/M checkpoint regulator, cells were exposed to talazoparib and CHK1 phosphorylation (Ser317) was examined by immunofluorescence and western blotting. In SGC7901 and BGC823 LV-Ctrl cells, CHK1 phosphorylation (Ser317) significantly increased after treatment with talazoparib. However, relatively reduced CHK1 phosphorylation (Ser317) was observed in response to talazoparib in LV-shMUS81 cells (**Figure 4A**).

CHK1 (Ser317) can be phosphorylated by ATR phosphorylation, and CHK1 phosphorylation prompts cells to enter G2M phase arrest for DNA repair (18–21). Therefore, whether MUS81 deficiency affected ATR activation was examined. Western blotting showed that MUS81 knockdown significantly decreased ATR activation and reduced the CHK1 activation response to talazoparib (**Figures 4B, C** **)**. These results indicated that MUS81 knockdown impaired the G2M phase checkpoint of cells by suppressing ATR/CHK1 signaling.

### MUS81 Knockdown Enhanced Anticancer Efficacy of Talazoparib in Gastric Cancer *In Vivo*

**Figure 5:**
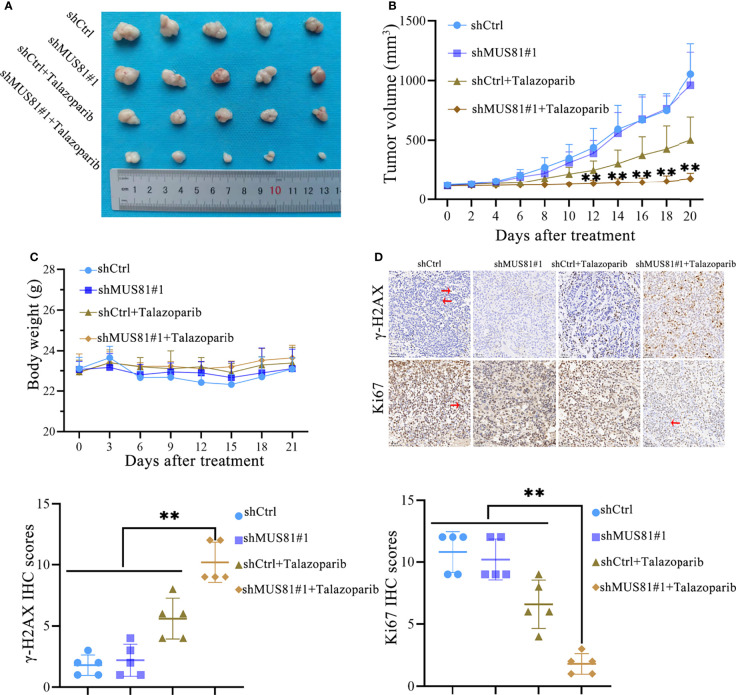
MUS81 knockdown enhances the anticancer efficacy of talazoparib in gastric cancer *in vivo*. **(A, B)** Tumor volume of each group on the indicated days of treatment (*t*-test). **P < 0.01. **(C)** Weight of nude mice on indicated days of treatment (*t*-test). **(D)** Representative images of γ-H2AX and Ki-67 immunohistochemistry (*t*-test). Positive expression (→). Negative expression (←). Magnification, 200×. **P < 0.01.

To demonstrate that MUS81 knockdown increases the antitumor effect of talazoparib *in vivo*, nude mice bearing MUS81-deficient and parental xenograft gastric cancer were treated daily with and without oral 0.33 mg/kg talazoparib. Talazoparib showed outstanding anticancer effect in the MUS81-deficient gastric cancer model (**Figure 5A**); the antitumor effect was most significant after 21 d of treatment (**Figure 5B**). There was no significant difference in the body weight among the four groups of nude mice (**Figure 5C**).

IHC studies indicated that after treatment with talazoparib, tumor tissues from the MUS81-deficient group showed lower Ki67 expression, suggesting lower proliferation ability compared to that of the parental xenograft group (**Figure 5D**). Furthermore, the expression of γ-H2AX was analyzed. As expected, MUS81 knockdown tumor cells showed higher expression of γ-H2AX than control cells in response to talazoparib (**Figure 5D**). Overall, these data represented excellent antitumor efficacy of talazoparib in the MUS81-deficient gastric cancer xenograft model.

### AZD5153 Sensitized the Anticancer Effect of Talazoparib in a MUS81-Dependent Manner in Gastric Cancer Cells

**Figure 6:**
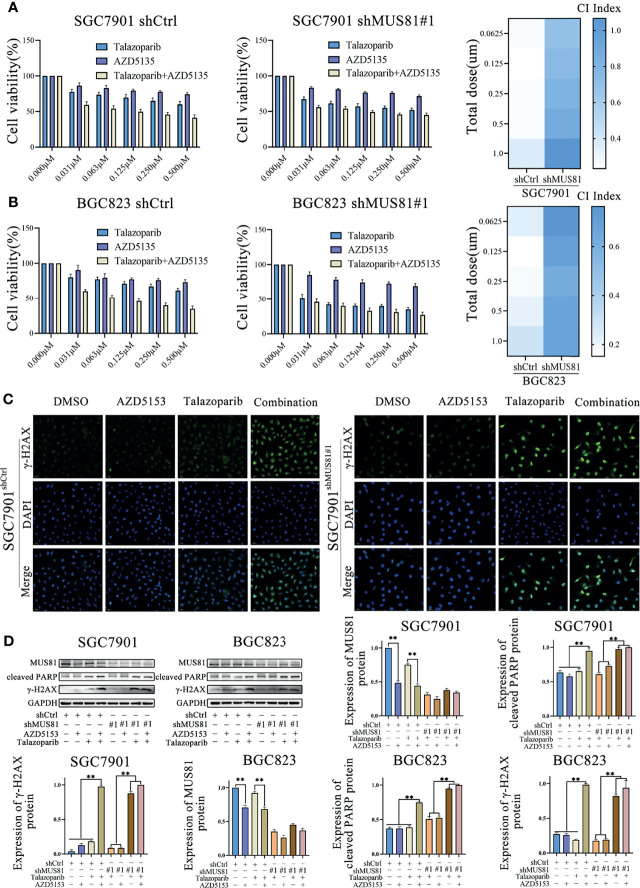
AZD5153 sensitizes the talazoparib anticancer effect in a MUS81-dependent manner in gastric cancer cells. **(A, B)** Viability of cells treated with monotherapy and combination therapy for 5 d, as determined using MTS (*t*-test). At five gradually increasing concentrations (0.0625 µmol/L to 1.000 µmol/L), the mean CI of SGC7901^shCtrl^ cells were 0.26, 0.23, 0.25, 0.26, and 0.36, respectively; the mean CI of SGC7901^shMU81^ cells were 0.69, 0.75, 0.81, 0.87, and 1.07, respectively; the mean CI of BGC823^shCtrl^ cells were 0.23, 0.15, 0.18, 0.21, and 0.29, respectively; the mean CI of BGC823^shMU81^ cells were 0.76, 0.69, 0.62, 0.67, and 0.67, respectively. The difference between shMU81 and shCtrl groups was statistically significant (all *P* < 0.01). **(C)** Immunofluorescence to detect the expression of phosphorylated checkpoint kinase 1 (p-CHK1) in cells treated with DMSO, AZD5153 (1 µmol/L), talazoparib (0.5 µmol/L), or combination therapy for 2 d. Magnification, 400×. **(D)** Western blot analysis examining the expression of MUS81, cleaved PARP, and γ-H2AX in gastric cancer cells after treatment with DMSO, AZD5153 (1 µmol/L), talazoparib (0.5 µmol/L), or combination therapy for 2 d (*t*-test). **P < 0.01.

Our previous study has shown that the BRD4 inhibitor AZD5153 downregulates MUS81 expression (10). Thus, it was hypothesized that AZD5153 might sensitize the talazoparib antitumor effect in gastric cancer cells by regulating MUS81 expression. To verify the potential synergy between AZD5153 and talazoparib, the effects of monotherapy and combination therapy were assessed in SGC7901 and BGC823 cell lines. MTS data showed that the AZD5153/talazoparib combination was synergistic with a combination index of less than 0.5 in LV-Ctrl cell lines. Interestingly, in the MUS81 knockdown cell line, the AZD5153/talazoparib combination effect was significantly impaired (**Figures 6A, B** **)**.

**Figure 7:**
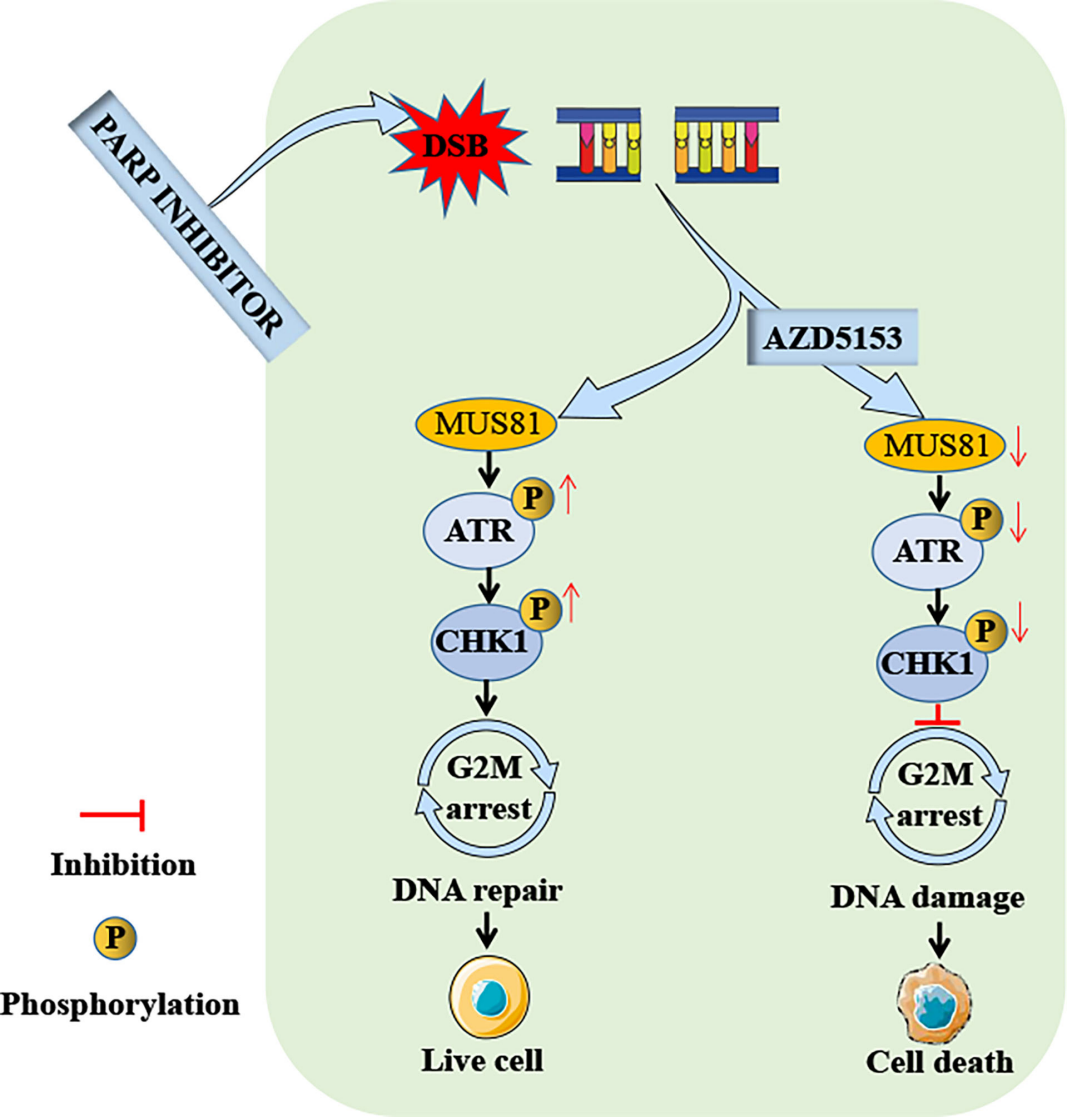
Schema of the antitumor effect of AZD5153 sensitizing gastric cancer cells to talazoparib. AZD5153 inhibits the ATR/CHK1 pathway activated by DNA damage by downregulating MUS81 expression, causing gastric cancer cells to continue mitosis with damaged DNA, ultimately leading to cell death.

Next, western blotting showed that AZD5153 significantly reduced MUS81 expression. Furthermore, similar to talazoparib therapy in MUS81 knockdown cells, the AZD5153/talazoparib combination therapy significantly improved the expression of γ-H2AX and cleaved PARP in LV-Ctrl cells (**Figures 6C, D** **)**. These data suggested that the efficacy of AZD5153 sensitized talazoparib in gastric cancer cells may be attributed to its ability to downregulate MUS81. AZD5153/talazoparib combination therapy promoted DNA damage and apoptosis in SGC7901 and BGC823 cells (**Figure 7**).

## Discussion

Among DNA damage repair pathways, PARP plays an indispensable role in the repair of DNA SSBs (22, 23). PARP inhibitors impair SSB repair to cause tumor cells to transform into DNA DSBs in the S phase, which leads to cell death due to mitotic catastrophe or apoptosis in tumors with BRCA1 and BRCA2 mutations as well as an HR deficiency (24). However, in gastric cancer, mutation rates in HRD genes (BRCA1 and BRCA2) are low, and individual PARP inhibitors have limited efficacy (25). Therefore, it is urgent to explore other targets to enhance the antitumor effect of PARP inhibitors and to expand the beneficiary population in gastric cancer. The DNA endonuclease MUS81 is a member of the endonuclear XPF family, which has been implicated in DNA repair *via* HR (7, 26–28). In the present study, MUS81 was involved in DNA damage repair and cell cycle regulation pathways in gastric cancer. MUS81 knockdown enhanced the cytotoxic effects of talazoparib-induced apoptosis in gastric cancer cells.

DNA damage checkpoints mainly include G1-S and G2-M, which regulate whether the cell continues mitosis or performs DNA repair or apoptosis (29, 30). Our research showed that talazoparib causes DNA damage in tumor cells, activates G2-M checkpoints, and leads to G2-M phase arrest. A previous study showed that MUS81 knockdown reverses the G2-M block caused by epirubicin in liver cancer cells (31). The current study also confirmed that MUS81 knockdown reversed G2-M block caused by talazoparib in gastric cancer, which might cause tumor cells to continue mitosis with unrepaired DNA and eventually lead to cell death. This research showed that talazoparib had significant antitumor effects in a MUS81-deficient SGC7901 xenograft model, and there was no significant difference in the body weight among the four groups of nude mice during the treatment.

In addition, it was observed that MUS81 knockdown reduced the activation of ATR and CHK1 in gastric cancer cells induced by talazoparib. ATR is one of the central regulators that control cellular responses to DNA damage (32, 33). In the G2 phase, activation of the ATR/CHK1 pathway blocks cells from continuing mitosis with damaged DNA (34, 35). Our previous study has shown that the CHK1 inhibitor LY2606368 impairs HR-mediated DNA damage repair, thereby sensitizing the antitumor efficacy of PARP inhibitors (36). Therefore, MUS81 knockdown promoted talazoparib-induced DNA damage by regulating the ATR/CHK1 pathway.

Overall, these findings indicated that sensitivity of gastric cancer to talazoparib relied on MUS81 inactivation. Our previous research has shown that AZD5153, a novel BRD4 inhibitor, downregulates MUS81 expression, and reduces the migration of gastric cancer cells *in vitro* and *in vivo* (10). Therefore, talazoparib combined with AZD5153 was applied to treat gastric cancer. In LV-Ctrl gastric cancer cells, combined MUS81 and AZD5153 treatment achieved the same antitumor effect as MUS81 knockdown combined with talazoparib treatment. Our previous work confirmed that AZD5153 inhibits the expression of sirt5 and impairs the MUS81 transcription function in gastric cancer cells (10); however, the underlying mechanism by which AZD5153 regulates MUS81 remains to be explored. In future, the combined use of AZD5153 and talazoparib in clinical trials may further verify their efficacy in the treatment of gastric cancer and may improve the prognosis of patients with advanced gastric cancer. However, *in vivo* experiments need to be conducted to examine whether AZD5153 can enhance the lethality of talazoparib to normal cells. Additionally, the combination therapy’s therapeutic window remains to be confirmed by further studies.

In conclusion, MUS81 was overexpressed in gastric cancer cells and was closely related to cell cycle regulation and DNA damage repair pathways. Moreover, MUS81 was a key molecule that enhanced the talazoparib antitumor effect in gastric cancer cells. MUS81 targeting markedly induced DNA damage and promoted cell apoptosis after talazoparib treatment by inhibiting ATR/CHK1 pathway activation. Importantly, AZD5153 enhanced the talazoparib antitumor effect in gastric cancer cells by reducing MUS81 expression. Taken together, talazoparib in combination with AZD5153 may be a promising treatment strategy for MUS81 overexpressed gastric cancer.

## Data Availability Statement

The datasets presented in this study can be found in online repositories. The names of the repository/repositories and accession number(s) can be found in the article/supplementary material.

## Ethics Statement

The animal study was reviewed and approved by the Institutional Animal Care and Use Committee of Tongji Medical College, Huazhong University of Science and Technology.

## Author Contributions

TW and PZ conducted the experiments, performed the data analysis and wrote the paper. CL, WL, QS, and LY performed the experiments. GX, JB, and RL analyzed data. YY and KT revised the manuscript and designed the experiment. All authors contributed to the article and approved the submitted version.

## Funding

This work was supported by the Natural Science Foundation of Hubei Province (No. 2019CFB660, 2019CFB100 and 2021CFB566), the Key Research and Development Program of Hubei Province (No. 2021BCA116) and the National Natural Science Foundation of China (No. 81874184, 82003205, and 82003131).

## Conflict of Interest

The authors declare that the research was conducted in the absence of any commercial or financial relationships that could be construed as a potential conflict of interest.

## Publisher’s Note

All claims expressed in this article are solely those of the authors and do not necessarily represent those of their affiliated organizations, or those of the publisher, the editors and the reviewers. Any product that may be evaluated in this article, or claim that may be made by its manufacturer, is not guaranteed or endorsed by the publisher.

## Abbreviations

ATR, ataxia telangiectasia and Rad3-related; BRCA, breast cancer susceptibility gene; CHK1, checkpoint kinase 1; DSB, double-strand break; HR, homologous recombination repair; PARP, poly (ADP-ribose) polymerase; SSB, single-strand break; TCGA, the Cancer Genome Atlas; Xpf, Xeroderma pigmentosum type F/Cockayne syndrome.
